# Beyond the Usual Suspects: Rare Causes of Hemoptysis

**DOI:** 10.3390/diagnostics16101465

**Published:** 2026-05-12

**Authors:** Ivana Sekulovic-Radovanovic, Ilya V. Sivokozov, Nensi Lalic, Spasoje Popevic

**Affiliations:** 1 Clinic of Pulmonology, University Clinical Center of Serbia, 11000 Belgrade, Serbia; spasapop@gmail.com; 2 Endoscopy Department, Central Tuberculosis Research Institute, 107564 Moscow, Russia; ilyavsivokozov@gmail.com; 3 Faculty of Medicine, University of Novi Sad, 21000 Novi Sad, Serbia; nenslialic@gmail.com; 4 Faculty of Medicine, University of Belgrade, 11000 Belgrade, Serbia

**Keywords:** hemoptysis, rare causes, vascular malformation, diffuse alveolar hemorrhage

## Abstract

Hemoptysis is a potentially life-threatening phenomenon with a wide range of underlying causes. While most episodes are linked to common conditions such as infections, malignancy, or pulmonary embolism, a proportion of cases are due to unusual and often unexpected etiologies. This narrative review summarizes published case reports, series, and observational studies describing rare causes of hemoptysis, including vascular malformations, congenital anomalies, benign tumors, systemic diseases, and unusual infections. These conditions are frequently overlooked, which may delay recognition and appropriate management. The reviewed examples highlight the variety of diagnostic challenges and the broad spectrum of therapeutic strategies that may be required, ranging from endovascular procedures and surgery to targeted medical therapy. Despite advances in diagnostic methods, a subset of patients remain classified as having idiopathic or cryptogenic hemoptysis. For this reason, clinicians should keep a broad differential diagnosis in mind and remain aware of rare but clinically important entities. Awareness of these uncommon presentations and individualized patient management are essential for improving outcomes and avoiding missed critical diagnoses.

## 1. Introduction

Hemoptysis refers to the coughing up of blood arising from the lower respiratory tract and represents a clinically significant symptom, with presentations ranging from mild, self-limited episodes to life-threatening massive bleeding. While the majority of cases are attributed to common causes such as bronchitis, infections (bronchiectasis, tuberculosis, aspergillosis), pulmonary embolism, treatment with antithrombotic agents and malignancies, a small subset of patients present with less frequent, often overlooked etiologies. Identifying these rare causes is essential for timely diagnosis and appropriate management, especially when routine investigations fail to reveal the source of bleeding [1,2,3].

This narrative review aims to provide a comprehensive overview of rare but clinically significant causes of hemoptysis. By synthesizing current literature and case-based evidence, we highlight unusual contributors to pulmonary bleeding and describe specific characteristics that may facilitate diagnosis. Recognizing these atypical presentations is essential for clinicians confronted with diagnostic uncertainty, and we hope to emphasize that raising awareness of rare entities can help ensure that no critical etiology is overlooked in the evaluation of hemoptysis. The rare causes of hemoptysis discussed in this review are summarized in Table 1.

A suggested diagnostic approach in patients with hemoptysis after exclusion of common causes is presented in Table 2.

## 2. Materials and Methods

This narrative review was conducted to identify and summarize published literature on rare causes of hemoptysis. A comprehensive search of PubMed and Google Scholar was performed from database inception through 30 August 2025, without language restrictions. The search strategy combined MeSH terms and free-text keywords, including “hemoptysis”, “rare causes”, “unusual etiology”, “vascular malformation”, “broncholithiasis”, “diffuse alveolar hemorrhage” and “tracheo-innominate fistula” among others. Reference lists of relevant articles and case reports were hand-searched to identify additional sources.

We included peer-reviewed case reports, case series, observational studies, and narrative or systematic reviews that addressed infrequent or atypical etiologies of hemoptysis. We excluded articles focused exclusively on common causes (e.g., tuberculosis, bronchiectasis, lung carcinoma) unless a rare differential was specifically discussed. Titles/abstracts were screened for relevance; full texts were reviewed when needed. Data extraction and thematic categorization were performed manually by the authors.

## 3. Hemoptysis Associated with Systemic Vascularization of the Lung

Systemic arterial vascularization of the lung is a rare congenital anomaly, considered as a part of the “sequestration spectrum,” but distinct in that the bronchial anatomy remains normal. Two main patterns are described: isolated systemic arterial supply to an otherwise normal lung and dual supply from both systemic and pulmonary arteries [4,5,6].

From an embryological standpoint, this anomaly is thought to arise from the failure of normal regression of early systemic arterial supply to the developing lung. During early stages of lung formation, the pulmonary parenchyma receives blood flow from multiple small branches originating from the dorsal aorta and related splanchnic vessels. As the pulmonary arterial system develops, typically around the sixth week of gestation, these primitive systemic vessels involute. However, in certain circumstances, such as impaired pulmonary perfusion or altered vascular development, this regression may be incomplete, resulting in persistent systemic pulmonary vascular connections after birth [7,8].

Although the descending thoracic aorta is the most frequent source, anomalous vessels may also originate from the abdominal aorta, celiac axis, intercostal arteries, or even the subclavian artery. These vessels are typically muscular at their systemic origin, but become elastic as they enter the pulmonary parenchyma. As a result, the intrapulmonary segment is exposed to systemic pressures, which over time may cause progressive vascular injury [9,10].

Clinically, patients may develop localized pulmonary hypertension, hemoptysis (ranging from intermittent, self-limiting hemoptysis to massive, life-threatening hemorrhage), dyspnea, or even high-output cardiac failure. Unlike classic sequestration, which presents with recurrent pulmonary infections due to abnormal bronchial communication, here the complications are primarily hemodynamic, driven by abnormal vascular load on normal lung parenchyma [7,8].

Anomalous arteries from the descending thoracic aorta most often supply the left lower lobe, while those arising from the abdominal aorta or celiac axis tend to supply the right lower lobe, usually in coexistence with normal pulmonary arterial branches. The left lower lobe is the most common site of involvement, but exceptionally rare cases have been documented. For example, Hwang et al. reported a case of a 29-year-old woman where an anomalous artery from the descending thoracic aorta supplied part of the left upper lobe, presenting with massive hemoptysis [11].

Previous reports have shown either surgical resection or embolization as standard management options. Surgical resection of the aberrant artery, often combined with lobectomy or segmentectomy of the affected lung, was the gold standard for treating anomalous systemic arterial supply to the lung. Embolization was reserved for patients considered inoperable, but for aberrant vessel diameter larger than 10 mm coil, embolization was not recommended because of the risk of incomplete occlusion and coil migration [12].

More recently, modern approaches have been described in the literature. Jiang et al. reported successful transarterial embolization using coils and vascular plugs, demonstrating the effectiveness of endovascular therapy as a minimally invasive option [13]. Sun et al. described coil embolization in an asymptomatic adult, resulting in complete devascularization and subsequent shrinkage of the involved lobe [14]. Yuan et al. published a case of massive hemoptysis treated with combined coil and glue embolization, with complete recovery and no recurrence during follow-up [15]. Han et al. presented thoracoscopic ligation of the aberrant systemic artery without lung resection, highlighting a less invasive surgical alternative that preserves pulmonary parenchyma [16]. Finally, Hatakeyama et al. advocated for a hybrid approach, combining preoperative embolization with thoracoscopic resection, which was shown to reduce intraoperative bleeding risk and improve safety in anatomically complex cases [17].

## 4. Congenital Pulmonary Vascular Anomalies and Hemoptysis

Hemoptysis may be linked in some instances to congenital anomalies involving either the pulmonary circulation or the cardiovascular system. Although these conditions are rare, they illustrate how developmental abnormalities can remain silent even for decades and later manifest with sometimes life-threatening bleeding. Among the reported entities, unilateral pulmonary artery hypoplasia or agenesis, pulmonary arteriovenous malformations (PAVMs), and hereditary hemorrhagic telangiectasia (HHT), represent the most clinically relevant causes described in the literature.

Unilateral pulmonary hypoplasia or agenesis is typically diagnosed in childhood and rarely recognized in adults. In such cases, pulmonary artery hypoplasia may lead to pulmonary hypertension and the development of systemic collaterals, which can be the cause of hemoptysis. Although exceptional, survival into adulthood without prior recognition of the condition is possible when compensatory mechanisms are present, making diagnosis challenging as it can easily mimic other pulmonary or cardiovascular diseases. For instance, Pinheiro et al. [18] documented an 84-year-old woman with left pulmonary artery hypoplasia who developed recurrent respiratory infections and later hemoptysis, reflecting the role of systemic collaterals and pulmonary hypertension in driving symptoms. In that case, management was conservative, with symptomatic treatment and close follow-up, as advanced age and comorbidities made surgical intervention unsuitable [18,19]. Another example is reported by Park et al. [20] showing the case of a 36-year-old man with isolated pulmonary artery hypoplasia and recurrent hemoptysis, in whom embolization of hypertrophied systemic collaterals was successfully performed, demonstrating that endovascular therapy may be an effective option in younger and otherwise fit patients.

PAVMs are rare vascular abnormalities defined by direct communication between the pulmonary artery and vein, creating a low-resistance right-to-left shunt. Most cases occur in the setting of HHT, accounting for nearly 70% of PAVMs, while 15–30% of patients with HHT develop PAVMs [21,22]. Although multiple lesions are typically associated with HHT, PAVMs may also arise in conditions such as hepatopulmonary syndrome, schistosomiasis, mitral stenosis, trauma, or chronic infections [23]. Clinical presentation ranges from asymptomatic findings to life-threatening bleeding; in HHT, spontaneous rupture may cause massive hemoptysis or hemothorax, reported in up to 8% of cases, with pregnancy increasing this risk [24]. Dramatic episodes are illustrated by case reports such as a 64-year-old woman with massive hemoptysis from a PAVM supplied by the internal mammary artery, ultimately requiring lobectomy. Early recognition and timely embolization remain crucial [25].

## 5. Systemic-Pulmonary Arterial Fistulas as Rare Sources of Hemoptysis

Systemic–pulmonary arterial fistulas (SA-PAFs) are uncommon connections between systemic arteries and the pulmonary circulation. They can be congenital or acquired (post-surgical, post-infectious, or post-traumatic). On chest computed tomography (CT) they are frequently misread as pulmonary arteriovenous malformations or even pulmonary embolism, typically appearing as aberrant pulmonary-artery filling defects. The internal mammary and intercostal arteries are most often involved. For hemoptysis work-up, contrast-enhanced chest CT can suggest SA-PAF and guide planning, but angiographic strategy matters: pulmonary arteriography alone may miss SA-PAFs because high systemic pressure limits opacification from the pulmonary side. As a result, these lesions are often underrecognized and underdiagnosed [26,27].

Across the literature, intercostal-to-pulmonary artery fistulas are reported most commonly in patients with prior TB or chronic airway infection, and they can present with anything from recurrent bleeding to sudden, life-threatening hemoptysis. One report details a 38-year-old man with post-tuberculosis bronchiectasis with clinically significant haemoptysis who was diagnosed with selective angiography which identified a fistulous connection from a right intercostal artery to tertiary pulmonary arterial branches, and coil embolization achieved durable hemostasis [28].

Kurt et al. reported a case illustrating how comorbidity can amplify risk. The patient, a 35-year-old man on hemodialysis and anticoagulation with a history of antituberculous therapy, presented with massive hemoptysis. CT angiography identified a fistula between the left eighth intercostal artery and branches of the left pulmonary artery. Initial hemostasis was achieved with selective endovascular embolization, but he subsequently developed pneumonia and recurrent hemoptysis. After multidisciplinary review, he underwent definitive surgical management with a left lower lobectomy [29].

Internal mammary-to-pulmonary artery fistulas have been reported as causes of hemoptysis in postoperative and infectious settings. Although it can be congenitally developed, most of the described cases are secondary to prior coronary artery bypass grafting, chest trauma, inflammatory conditions, chronic infections, or neoplasia. In mildly symptomatic cases, conservative therapy may be appropriate; however, patients with persistent or severe bleeding, or those who are poor surgical candidates, are typically managed percutaneously with coil embolization, covered stents, or liquid embolic agents, approaches that have shown good clinical effectiveness [30,31].

A coronary-bronchial artery fistula is a rare vascular communication that is often silent, but in patients with chronic airway disease, especially bronchiectasis, it may present with hemoptysis and ischemic chest pain from coronary steal. ECG-gated, contrast-enhanced CT angiography usually delineates the course and drainage better than routine coronary angiography and is the preferred noninvasive test. When there is active bleeding or a high-flow shunt, endovascular embolization is generally the treatment of choice. In the case reported by Lee et al., a patient with bronchiectasis presented with chest pain and hemoptysis; CT identified a coronary–bronchial connection, and targeted embolization successfully controlled the bleeding with resolution of symptoms [32].

To conclude this chapter, it is important to highlight aortobronchial fistula, a rare and potentially fatal entity that most often follows rupture of a thoracic aortic pseudoaneurysm. Etiologies include atherosclerosis, infection (notably tuberculosis), trauma, and prior interventions such as aortic surgery, coarctation repair, endovascular aortic procedures, and esophagectomy. Clinically, patients may present with massive hemoptysis, leading to acute respiratory failure, hypotension, and death without immediate treatment. Thoracic endovascular aortic repair (TEVAR) is an intervention of choice and generally preferred as the initial (and often definitive) intervention, given it is safer, less invasive, compared with open surgical repair [33,34].

## 6. Rasmussen Aneurysm

Rasmussen’s aneurysm represents an inflammatory pseudoaneurysmal dilatation of a pulmonary artery branch situated adjacent to a tuberculous cavity. It occurs in a subset of such cavitary lesions, estimated at around 5%, and carries the risk of rupture, which can result in massive, potentially fatal hemoptysis [35,36]. Structural changes in the vessel wall occur during the healing phase of the disease, with both the adventitia and media being replaced by granulation tissue and subsequently by fibrin. These changes result in thinning of the arterial wall, segmental pseudoaneurysm formation, and eventual rupture. Lesions are most commonly located in the upper lobes and tend to have a peripheral distribution [37].

Hemoptysis is a frequent clinical manifestation of pulmonary tuberculosis; however, massive hemoptysis occurs in only about 8% of cases and carries a mortality rate ranging from 5% to 25% [38]. Massive hemoptysis most often results from erosion of the pulmonary, bronchial, or intercostal arteries. The preferred imaging modality for evaluating Rasmussen’s aneurysm is CT pulmonary angiography, which, with the use of intravenous contrast, can demonstrate focal dilatation of the pulmonary artery branches. Due to its low incidence, however, Rasmussen’s aneurysm may be easily overlooked on radiologic assessment [39].

Management can be difficult, as the patient’s condition may worsen even with appropriate treatment. Endovascular therapy, most often transarterial catheter embolization, is generally preferred as the first line management. Techniques include glue embolization, coil packing, gel foam, detachable balloons, or placement of a stent graft [40]. In cases refractory to angiographic embolization, surgery may be considered as an alternative; however, emergency resection performed during active massive hemoptysis carries a high risk of morbidity and mortality [41].

Through reviewed case reports and case series, Rasmussen’s aneurysms have been documented in a range of sizes and anatomical locations, findings that may influence both clinical presentation and management strategies. An illustrative example is provided by Oukrid et al. [42] who reported a 53-year-old man with Down syndrome and a history of treated pulmonary tuberculosis that developed bilateral upper-lobe Rasmussen aneurysms. CT pulmonary angiography revealed cavitary lesions containing endocavitary pseudoaneurysms. Despite timely diagnosis, the patient experienced sudden massive hemoptysis within hours and died. The authors emphasized that this complication should be considered in patients with cavitary pulmonary lesions and hemoptysis, and that early imaging combined with prompt endovascular management is critical to preventing fatal outcomes following aneurysm rupture.

## 7. Hughes–Stovin Syndrome

Hughes–Stovin syndrome (HSS) is an exceptionally rare disorder marked by extensive venous thrombosis in combination with multiple pulmonary and/or bronchial artery aneurysms. It is frequently regarded as a vascular variant of Behçet’s syndrome, also known as the “Silk Road disease.” The clinical course is typically described in three stages: initial thrombophlebitis, subsequent development of pulmonary or bronchial aneurysms, and eventual aneurysmal rupture, often presenting with hemoptysis. Hemoptysis results from the rupture of dysplastic vessels [43,44]. Pulmonary artery aneurysms (PAA) are rare vascular abnormalities that can remain clinically silent but carry the risk of rupture or dissection, and in some cases may have an underlying genetic predisposition [44,45]. Diagnosis is typically established by CT pulmonary angiography, which demonstrates aneurysmal dilatation often accompanied by adherent in situ thrombosis, consistent with the diagnostic criteria proposed by the HSS International Study Group [46].

In a systematic review of 57 published cases of HSS, Emad et al. reported that most patients were male (75.4%). Hemoptysis was observed in 42.1% of patients at the time of initial presentation and eventually occurred in up to 93% over the course of the disease, with a mortality rate of 21.1%. Pulmonary artery aneurysms were bilateral in most cases (93%) and were most commonly found in the lobar (87.7%) and segmental (73.7%) branches. Fatal outcomes were more frequent in patients with inferior vena cava thrombosis and ruptured aneurysms, whereas treatment with corticosteroids, cyclophosphamide, azathioprine, or combined immunomodulatory regimens was associated with improved survival. These findings emphasize that in HSS, hemoptysis is both a common and potentially lethal manifestation, typically resulting from ruptured pulmonary artery aneurysms, and that timely immunosuppressive therapy can be life-saving [46].

A recent case report by Manole et al. [44] further underlines the need to consider Hughes–Stovin syndrome in the differential diagnosis of hemoptysis, particularly when endobronchial findings mimic tumor. The authors described a 35-year-old male in whom CT imaging showed a lower lobe lung mass initially suspected to be a vascularized bronchial tumor. Bronchoscopy revealed a vegetating, hemorrhagic lesion obstructing a segmental bronchus, with significant bleeding during biopsy. Subsequent PET-CT confirmed the lesion as an aneurysmal dilatation of the inferior left lobar pulmonary artery. The patient was HLA-B51 positive, and the aneurysm regressed completely under corticosteroid and cyclophosphamide therapy. This case highlights that endobronchial appearance alone may be misleading, and a high index of suspicion is essential to avoid hazardous biopsy in vascular lesions.

## 8. Diffuse Alveolar Hemorrhage

Diffuse alveolar hemorrhage (DAH) is a clinicopathologic entity in which injury to the pulmonary microvasculature leads to the accumulation of blood within the alveolar spaces. The characteristic clinical constellation includes hemoptysis, anemia, and diffuse pulmonary infiltrates, often with hypoxemic respiratory failure. Although hemoptysis is the most common manifestation, it can be initially absent at presentation in up to one-third of cases only to develop later in the course of illness, making diagnosis challenging. Symptoms such as cough, dyspnea, chest pain, or fever are nonspecific, and DAH may occur at any age. Flexible bronchoscopy with bronchoalveolar lavage is the main diagnostic method: sequential BAL samples typically show an increasing red blood cell count, confirming intraalveolar bleeding, although it is not the underlying cause [47]. DAH can be classified into three main histopathologic categories: pulmonary capillaritis, bland pulmonary hemorrhage, and diffuse alveolar damage-associated hemorrhage. Etiologically, DAH can be broadly divided into immune-mediated and non-immune causes. Immune-mediated forms are most often associated with anti-neutrophil cytoplasmic antibody-associated vasculitides and other systemic autoimmune diseases, including small-vessel vasculitis and connective tissue disorders. In contrast, non-immune causes include a range of conditions, particularly infections, inhalational injury, complications related to bone marrow transplantation, and other less common entities such as idiopathic pulmonary hemosiderosis. Because of its unpredictable course and life-threatening potential, DAH can only be effectively managed by revealing the underlying cause [47,48].

In the following section, we will highlight some of the less common and diagnostically challenging forms of DAH.

### 8.1. Idiopathic Pulmonary Hemosiderosis

Idiopathic pulmonary hemosiderosis (IPH) represents a rare cause of DAH. It is typically characterized by repeated episodes of hemoptysis, accompanied by pulmonary infiltrates on imaging and the development of iron deficiency anemia. Hemoptysis is the initial symptom in approximately 80% of patients, and the disease course is usually marked by periods of worsening alternating with phases of clinical stability. The clinical presentation may range from mild blood-streaked sputum to massive hemorrhage with acute respiratory failure. In some patients, hemoptysis may persist over time without complete resolution. The etiology and pathogenesis remain poorly understood, and no definitive immunologic mechanism has been established. Diagnosis is supported by the presence of hemosiderin-laden macrophages in bronchoalveolar lavage, but confirmation generally requires transbronchial or surgical lung biopsy. On lung biopsy, findings typically include intraalveolar hemorrhage with accumulation of hemosiderin-laden macrophages, in the absence of vasculitis or immune complex deposition [49,50].

### 8.2. Targeted and Immuno-Oncologic Therapies

In the era of expanding use of innovative cancer therapies, it is important to keep in mind that these agents can provoke severe pulmonary complications such as DAH. Bevacizumab, an anti-vascular endothelial growth factor (VEGF) monoclonal antibody, exerts its antitumor effect by inhibiting angiogenesis—regressing existing vessels and preventing the formation of new ones [51,52]. Hemorrhagic events have been documented across multiple organ systems as adverse effects of bevacizumab; however, pulmonary manifestations are particularly severe, more difficult to manage, and carry a higher risk of fatal outcomes. As a result, bevacizumab has been repeatedly linked with pulmonary hemorrhage and hemoptysis, which, although rare, represent some of the most serious complications of therapy. Hu et al., in a large FAERS-based analysis, emphasized that these events may not be the most frequent but carry the highest clinical risk, underlining the need for close monitoring and timely intervention. The combination of BV with chemotherapy showed a significantly higher reporting risk for pulmonary hemorrhage and hemoptysis compared with BV monotherapy. Given the widespread use of bevacizumab in oncology, both as monotherapy and in combination regimens, awareness of these risks is essential in routine practice [53].

Immune checkpoint inhibitors such as nivolumab and pembrolizumab have also been associated with DAH. Through literature reports, numerous cases have been documented where DAH developed as an adverse effect of this type of therapy, with highly variable clinical outcomes. Sugano et al. described a patient who developed DAH after four cycles of pembrolizumab, confirmed by BAL, who was successfully treated with corticosteroids and fully recovered, although pembrolizumab therapy was discontinued [54]. Another case illustrated a different course, where nivolumab-induced DAH presented with bloody sputum and ground-glass opacities mimicking pseudoprogression; bronchoscopy again confirmed the diagnosis, and the patient improved after corticosteroids, allowing continuation of oncology treatment [55]. In contrast, a fatal case of DAH was reported following nivolumab monotherapy in a 60-year-old man with metastatic bladder cancer, who developed rapidly progressive dyspnea and dry cough without hemoptysis. CT initially showed subtle bilateral ground-glass opacities that progressed to diffuse infiltrates and consolidation. Bronchoscopy with BAL revealed increasingly hemorrhagic aliquots, and autopsy confirmed extensive alveolar hemorrhage without other lung pathology—all in the absence of bleeding diatheses or hemoptysis, culminating in refractory respiratory failure and death [56].

Combination therapy can further increase the risk of developing DAH. In a published report, DAH occurred after three months of dual checkpoint blockade with ipilimumab and nivolumab, presenting with hemoptysis and dyspnea; bronchoscopy demonstrated gross hemorrhage consistent with DAH [57].

All these examples underline that novel targeted and immunomodulatory therapies, though transformative in oncology, may trigger DAH—with or without hemoptysis—and bronchoscopy often proves decisive for establishing the diagnosis. This is a complication that clinicians should keep in mind and actively exclude in the diagnostic work-up, especially since it may initially present without hemoptysis.

### 8.3. Drug-Induced Lung Injury

Drug-induced lung injury has been linked to the inhalation of illicit substances. The best-described manifestation in the literature is “crack lung”, an acute pulmonary syndrome characterized by diffuse alveolar damage and hemorrhagic alveolitis occurring within 48 h of cocaine or other drug inhalation [58,59].

Patients can present at any age depending on prior drug use, most often arriving at the emergency department with acute hemoptysis, chest pain, cough, and respiratory distress. Because the clinical presentation often mimics pulmonary embolism, many patients undergo CT pulmonary angiography. While embolism is usually excluded, imaging may demonstrate diffuse infiltrates, bronchial wall thickening, ground-glass opacities, or other nonspecific findings. Bronchoscopy provides both diagnostic and therapeutic value, particularly in ruling out infection and malignancy as potential causes of this clinical presentation [60,61].

Although no formal guidelines exist, individual case reports suggest that systemic corticosteroid therapy may be beneficial. Regular follow-up is recommended, particularly in chronic users, due to the risk of persistent parenchymal injury. Case reports by Reichert et al. [60], Giacomi et al. [59], and Jiménez-Zarazúa et al. [62] describe varying severity and outcomes following drug inhalation, but all emphasize that timely recognition and a high index of suspicion are essential for rapid diagnosis and successful treatment.

## 9. Burn-Injury Related Hemoptysis

Inhalation injury represents acute damage to the respiratory tract caused by smoke, steam, or toxic gases. Although it is most often seen together with cutaneous burns, it can also occur in patients who have no visible external injuries, which makes early recognition very important. This condition is more likely to occur in house fires, explosions, or vehicle accidents, but it has also been reported in open-space accidents. The primary damage usually affects the airway mucosa, but in severe cases it can extend to the lower tracheobronchial tree and even the lung parenchyma. Clinical manifestations range from mild nasopharyngeal irritation, cough, and hoarseness to stridor, dyspnea, chest discomfort, and hemoptysis. Hemoptysis is a rare but possible complication of smoke inhalation, especially when mucosal injury progresses to deeper tissue damage. A key diagnostic challenge arises in patients who were only exposed to smoke inhalation without cutaneous burns, as their injury may initially go unrecognized. In all cases, it is important to emphasize that early bronchoscopic evaluation is crucial to assess the extent of airway damage, predict disease progression, and guide timely management [63,64,65].

## 10. Bronchial Dieulafoy’s Disease

Dieulafoy’s disease is an extremely rare vascular anomaly, initially described in the gastrointestinal tract and characterized by abnormally large-caliber submucosal arteries that are prone to erosion and bleeding [66]. Bronchial Dieulafoy’s disease (BDD) was first reported in 1995 by Sweerts et al. [67], who described two patients presenting with massive hemoptysis, both of whom were successfully treated by lobectomy. Histological examination in both cases revealed a large-caliber artery with focal thinning of the tunica media, extending between the bronchial cartilages into the submucosa, or passing through the mucosa to open into the bronchus, identical to the lesion known as Dieulafoy’s disease of the gastrointestinal tract.

The exact etiology of BDD remains uncertain. Proposed mechanisms include congenital vascular malformations and chronic bronchial wall damage as a consequence of prior pulmonary infections. Some reports suggest that patient age and a history of tobacco use may contribute to its development [67,68,69]. In a retrospective analysis of 73 patients with BDD, Qian et al. found a clear male predominance (M:F ≈ 2:1) and a mean age of 47.2 years (range 8 months–85 years). Lesions were most often located in the right bronchial tree (73.6%), predominantly in the right lower, middle, and upper lobes (30.6%, 22.2%, and 20.8%, respectively). All patients presented with hemoptysis, which was massive in 68.5% of cases. Clinically, hemoptysis represents the predominant manifestation. On bronchoscopy, BDD typically appears as a small, sessile, nonpulsating mucosa-covered lesion with a characteristic whitish cap [70,71]. Although histologic confirmation is desirable, biopsy should be avoided when BDD is suspected due to the high risk of severe bleeding [72]. Endobronchial ultrasound (EBUS) may assist in assessing suspicious lesions during bronchoscopy [73]. In a case reported by Ganganah et al. [74], EBUS performed for suspicious bronchial lesions revealed features suggestive of vascular structures (two circular anechoic areas with a hyperechoic border), leading to avoidance of biopsy and confirmation of an aberrant bronchial artery on further imaging, followed by successful embolization.

Although no standardized diagnostic criteria exist, arteriography can sometimes reveal vascular anomalies that, in the right clinical context, support the diagnosis. Selective arterial embolization is often used as initial therapy, but its long-term efficacy is limited, and surgery is frequently required [69].

## 11. Hemoptysis Associated with Tracheostomy

Tracheostomy is a procedure that involves creating an opening through the neck and the anterior wall of the trachea to secure the airway. It can be performed either as an open surgical tracheostomy or as a percutaneous dilational tracheostomy. Indications for this intervention may vary, but the most common is prolonged mechanical ventilation in the setting of respiratory failure. A tracheostomy can be temporary or permanent, depending on the underlying condition [75]. As with any invasive procedure, tracheostomy carries certain risks and potential complications, which will be discussed in the following section.

### 11.1. Tracheo-Innominate Fistula

Tracheo-innominate fistula (TIF) is a very rare but potentially fatal complication of tracheostomy, with reported incidence between 0.1% and 1%. It can manifest with different degrees of bleeding, clinically presenting as hemoptysis, peristomal bleeding, or hemorrhage through the tracheostomy tube, and carries an extremely high risk of mortality. Although most frequently described following tracheostomy, TIF has also been reported after tracheal resection and reconstruction, penetrating neck trauma, migration of adjacent orthopedic hardware, and, rarely, after the placement of endovascular stent grafts [76,77,78]. In some cases, sentinel bleed, characterized by minor bleeding that spontaneously ceases, may precede sudden massive hemorrhage [75].

The innominate artery usually crosses the trachea between the 6th and 9th cartilage rings, which explains the increased risk of TIF when the tracheostomy is placed below the third tracheal ring. Additional risk factors include cuff over-inflation causing pressure necrosis, tracheitis, prolonged intubation, use of corticosteroids or immunosuppressants, prior neck irradiation, recurrent hypotension, anomalous arterial anatomy, and local infection at the stoma site [79,80].

The main diagnostic modalities include bronchoscopy, conventional angiography, and computed tomography angiography; all of them can assist in identifying the bleeding source, but all have limited sensitivity. Clinical suspicion remains critical. Bronchoscopy may directly demonstrate bleeding on the anterior tracheal wall, while angiography and CT angiography can show extravasation from the innominate artery into the airway [80,81].

Management of TIF requires fast recognition and immediate action, starting with temporary hemostasis measures such as the Utley maneuver or cuff overinflation, followed by definitive treatment either through open surgical repair (most commonly ligation of the innominate artery with tracheal coverage) or endovascular stent placement in high-risk patients [80].

### 11.2. Other Tracheostomy-Related Vascular Fistulas: Carotid-Tracheal Fistula

Although TIF remains the mostly described vascular complication of tracheostomy in the available literature, it is important to remember that other major vessels may also be affected, leading to fistula formation and potentially fatal hemorrhage, for example, carotid artery involvement.

Shylendran et al. [82] reported the case of a 64-year-old female who developed massive bleeding approximately two weeks after tracheostomy. Computed tomography angiography (CTA) demonstrated a small pseudoaneurysm of the right common carotid artery, arising just distal to its origin from the brachiocephalic artery, with fistulization into the anterior tracheal wall and the tracheostomy tube in situ. This case highlighted the role of CTA in both establishing the diagnosis and planning endovascular treatment.

### 11.3. Hemorrhage Associated with Granulation Tissue Formation

One of the complications of tracheostomy is the development of tracheal granulation tissue, which can develop both early in the post-interventional period or later during follow-up period. Although frequently asymptomatic, excessive granulation can lead to clinically significant problems such as airway obstruction or hemoptysis and should be considered in the differential diagnosis of these conditions in patients with a history of tracheostomy. Medrek et al. [83] described a case of a 45-year-old man who was presented with hemoptysis four months after decannulation. Bronchoscopic evaluation confirmed the presence of granulation tissue, which was removed using an electrocautery snare, resulting in complete resolution of hemoptysis. The authors also emphasized the importance of surveillance and timely recognition of this complication [83,84].

## 12. Pulmonary Hamartomas as Rare Cause of Hemoptysis

Hamartomas are the most common type of benign lung tumor. They are composed of bronchial epithelium, cartilage, mesenchymal connective tissue, and fibroblastic elements. In most cases, pulmonary hamartomas are asymptomatic, peripherally located, measure less than 2 cm in diameter, and are detected incidentally during radiological evaluation. On rare occasions, they may reach a larger size. In endoluminal hamartomas and those of greater dimensions, hemoptysis and irritative cough may occur as the initial manifestations of the disease. Case reports in the literature describe instances where even massive hemoptysis was among the first symptoms, particularly in larger hamartomas, due to their impact on the airways and pulmonary vasculature. Because of the atypical clinical presentation and the sometimes extensive endoscopic appearance, such lesions can be mistaken for malignancy. Chest computed tomography plays a key diagnostic role by providing detailed information on the lesion’s structure and guiding further management. The primary treatment is surgical resection; however, for smaller endoluminal lesions, bronchoscopic techniques such as YAG laser, electrocautery, cryotherapy, or argon plasma coagulation can be performed. After finalizing the treatment, complete resolution of symptoms is typically achieved [85,86,87,88,89,90,91].

## 13. Foreign Body-Associated Hemoptysis

Foreign body aspiration into the tracheobronchial tree is far less common in adults than in children. Both the literature and clinical practice describe a wide range of aspirated objects, from soft food particles to solid animal or fish bones, organic materials, dental prostheses or teeth, as well as various metallic and plastic objects such as syringe caps. In many cases, diagnosis requires a high index of clinical suspicion, and the identification of foreign bodies in the tracheobronchial tree is often delayed or initially overlooked, which may result in chronic symptoms and subsequent complications. A diagnostic challenge also arises from the fact that many foreign bodies are not visible on routine chest radiography. Without precise information that something has been aspirated, diagnosis can be delayed, especially given the nonspecific symptoms that occur in many lung diseases. Obtaining a detailed medical history is crucial, particularly in identifying predisposing risk factors for aspiration such as impaired consciousness, neuromuscular disorders, dysphagia, alcohol or drug abuse, altered mental status, as well as structural or functional abnormalities of the upper aerodigestive tract, including those occurring after radiotherapy for head and neck cancers [92,93,94].

The clinical presentation depends on the location and duration of impaction, as well as the characteristics of the aspirated object, including its size, material, and shape. The most common symptoms are persistent cough, dyspnea, and wheezing, while hemoptysis may also occur, occasionally even in massive forms. It is important to note that in some cases, foreign body aspiration can cause severe respiratory distress and, in rare instances, even fatal outcomes [92,95].

Soto et al. [96] reported an unusual case of a foreign body that was not aspirated but rather retained postoperatively. The patient had a gossypiboma (retained surgical gauze/sponges) following an emergent thoracotomy for penetrating trauma. In this rare occurrence, the gossypiboma eroded from the pleura into the airway, presenting with productive cough and intermittent hemoptysis. Chest CT demonstrated findings consistent with a foreign object, and surgical resection was required.

Chest CT can be a valuable diagnostic tool in suspected foreign body aspiration, but bronchoscopy remains the gold standard for both diagnosis and treatment. Flexible bronchoscopy provides real-time visualization of the airways, allowing direct inspection and retrieval of the foreign body. Endoscopic findings vary depending on the duration of retention and may occasionally mimic an endoluminal tumor. Hemoptysis, which can range from mild to severe, is one of the most clinically significant complications associated with retained foreign bodies. Its presence often reinforces the need for urgent bronchoscopic intervention. While flexible bronchoscopy is usually sufficient, extraction may be technically demanding in anatomically complex locations or in patients with underlying pulmonary disease. In such cases, rigid bronchoscopy offers a larger working channel, better control of the airway, and the ability to manage complications such as significant bleeding. Timely removal is essential to prevent long-term sequelae, including granulation tissue formation, bronchiectasis, lung abscess, and bronchial stricture [92,97,98,99].

## 14. Broncholithiasis

Broncholithiasis is a condition characterized by the presence of calcified or ossified material, referred to as broncholiths, within the tracheobronchial tree. Broncholiths are typically composed of calcium phosphate (approximately 85–90%) and calcium carbonate (about 10–15%), resembling the mineral composition of bone [100]. Depending on their anatomical relationship to the tracheobronchial tree, broncholiths can be classified as endobronchial, peribronchial, or transbronchial [101].

The exact incidence and prevalence of broncholithiasis remain unknown. It is considered that any focal process that can lead to dystrophic calcification may also result in broncholithiasis. Several hypotheses have been proposed regarding the formation of broncholiths, but none fully explains the underlying mechanism. What is known is that broncholith development is most often associated with tuberculous infections, histoplasmosis, and other infections such as cryptococcosis, coccidioidomycosis, endobronchial aspergillosis, endobronchial nocardiosis, and *Mycobacterium kansasii*. Other non-infectious causes include malignancy, calcified retained foreign bodies, and erosion by calcified bronchial cartilage [102].

Although some patients may be asymptomatic, the clinical presentation can vary. The most common symptoms are cough and hemoptysis, followed by chest pain, wheezing, fever, and dyspnea. Less common manifestations include massive hemoptysis and acute airway obstruction. Lithoptysis is rare [101,102].

Hemoptysis is a common clinical presentation and also one of the complications of broncholithiasis. In most cases, it results from irritation of the bronchial wall, while in rare instances it may lead to vascular injury and development of bronchovascular fistulas, which can cause potentially fatal bleeding [102,103].

CT scanning is often preferred over plain chest radiography when broncholithiasis is suspected. It allows precise assessment of the relationship between broncholiths and adjacent bronchi, as well as a detailed view of airway anatomy, including possible deformities such as “bronchial twist.” CT can also demonstrate the absence of a soft tissue mass, which helps distinguish it from fibrosing mediastinitis, most commonly associated with *Histoplasma capsulatum* infection, but also reported in association with sarcoidosis. In addition, it can reveal calcifications with an endobronchial component or those located peribronchially with signs of airway compression. Most importantly, CT enables a thorough evaluation of the broncholith–bronchial wall relationship, which is a key factor in determining the appropriate management strategy [101,102,104,105].

Bronchoscopy plays a key role in both the diagnosis and management of broncholithiasis. Endoscopic findings vary; in addition to visualizing the broncholith itself, common observations include mucosal edema and hyperpigmentation, bleeding, granulation tissue adjacent to the broncholith, and airway distortion. Bronchoscopy enables direct evaluation of the airways, assessment of their relationship to the broncholith, and detection of any fistulae, all of which are significant in determining further management strategies [101,102,106].

According to the review by Alshabani et al. [102], there are no established guidelines for the optimal management of broncholithiasis. Initial bronchoscopy is generally recommended for all patients, primarily to assess airway anatomy and the relationship of the broncholith to the bronchial wall. This evaluation guides the decision between endoscopic intervention and surgical management. Bronchoscopic removal should be performed with extreme caution by an experienced bronchoscopists with thoracic surgical backup available. Forceful extraction carries a high risk of massive hemorrhage or airway wall injury. Any significant bleeding during the procedure warrants immediate reassessment. Surgical removal is typically advised for peribronchial and transbronchial broncholiths that are firmly attached to the airway wall or only partially protrude into the lumen, where endoscopic extraction is unlikely to be safe or successful. Other therapeutic approaches for managing broncholithiasis described in the literature include broncholithotripsy using electrohydraulic and pulsed-dye laser lithotripters via a rigid bronchoscope, as well as laser techniques such as Neodymium:Yttrium–Aluminum–Garnet (Nd:YAG) laser and Holmium:Yttrium–Aluminum–Garnet (Ho:YAG) laser.

## 15. Lipoid Pneumonia

Lipoid pneumonia is a rare and probably underdiagnosed disease, with its true incidence remaining unknown. It is usually classified into exogenous and endogenous forms, depending on the origin of lipids deposited in the lungs. Endogenous lipoid pneumonia develops as a result of impaired function of monocytes and macrophages, leading to recurrent infections and cholesterol accumulation due to tissue destruction and inadequate repair. It most commonly occurs as a postobstructive bronchial condition and has been reported in association with various pulmonary diseases. Exogenous lipoid pneumonia arises from aspiration of lipids, most frequently mineral oils or liquid paraffin used as laxatives, but it has also been described in fire-eaters exposed to kerosene and similar hydrocarbons. Patients are often unaware of the aspiration of oil-based agents, as these fatty substances do not stimulate the cough reflex. Rare idiopathic cases have also been reported [107,108,109].

The most frequently affected sites are the middle lobe, followed by the right lower lobe, left lower lobe, and lingula [110]. Imaging findings on chest radiographs and CT scans often include ground-glass opacities, diffuse pulmonary microcalcifications, and pronounced thickening of the interlobular septa. Exogenous lipoid pneumonia may appear either as a consolidation or as a solitary pulmonary nodule [111].

Most cases of lipoid pneumonia are asymptomatic. When present, symptoms may include chronic cough, dyspnea, chest pain, pleural effusion, and, more rarely, hemoptysis, which has been documented in several reported cases throughout the literature. The disease may progress to chronic respiratory impairment. Because of the nonspecific nature of symptoms and radiographic findings, diagnosis is often challenging. In patients with chronic cough and hemoptysis, the differential diagnosis is broad, and diffuse lesions can easily be misinterpreted as malignant disease, for example mimicking invasive adenocarcinoma, as described in the literature by Chieng et al. [109,112].

After radiological findings, the diagnosis is usually confirmed by histopathological examination, most often through bronchoscopy. Bronchoalveolar lavage (BAL) fluid typically contains numerous lipid-laden macrophages, while transbronchial lung biopsy specimens reveal clusters of foamy macrophages within the alveolar spaces, often accompanied by granulomatous lesions. Additionally, the BAL fluid may spontaneously separate into an oily upper phase and a lower aqueous phase. In certain cases, however, the diagnosis can only be established through open lung surgery, as illustrated in the case report by Simmons et al., where negative bronchoscopic findings and insufficient information regarding the patient’s medication history delayed recognition. The authors also emphasized the importance of clinical awareness, particularly in elderly patients and those with gastroesophageal reflux disease or swallowing difficulties who are prescribed mineral oils [108,109,110,113].

For exogenous forms, the avoidance of further exposure and the use of corticosteroids combined with lavage are considered the mainstays of treatment. It is important to exclude mycobacterial infection before initiating corticosteroid therapy, given its well-documented association with lipoid pneumonia [108,109,110].

## 16. Pulmonary Endometriosis

Thoracic endometriosis syndrome (TES) refers to the presence of ectopic endometrial-like tissue within the thoracic cavity, including the bronchial tree, lung parenchyma, pleura, and diaphragm. It may manifest through several clinical patterns, most commonly as catamenial pneumothorax, followed by catamenial hemothorax, hemoptysis, or pulmonary nodules. Symptoms typically follow a catamenial pattern, appearing from up to 24 h before to 72 h after the onset of menstruation. While pleural implantation is most common overall, in cases of thoracic endometriosis, catamenial hemoptysis occurs more frequently than pneumothorax [114,115].

Thoracic endometriosis is a rare disease with unclear causes and pathogeneses affecting 2–10% of women [116]. Although rare, it represents an important and often under-recognized cause of recurrent hemoptysis in women of reproductive age.

TES should be considered in the differential diagnosis of any woman of reproductive age presenting with chest or scapular pain, cough, hemoptysis, or dyspnea that worsens during menses. This is particularly relevant in patients with a history of pelvic endometriosis, which is associated with TES in approximately 53% of cases, with a mean interval of 5 years between diagnoses. The thorax represents the most common site of extra-pelvic endometrial implantation [114,117,118].

The mechanism of distant dissemination of endometrial cells remains incompletely understood. Several theories have been proposed, yet none alone can account for all clinical manifestations of the syndrome, supporting the concept that the etiology of TES is multifactorial. One suggested mechanism involves hematogenous or lymphatic spread, particularly when explaining the occurrence of bronchopulmonary endometrial lesions. The predominance of right lung involvement has been attributed to the more extensive lymphatic drainage on the right side of the diaphragm [115,119,120].

Chest CT, though not specific, is regarded as the preferred imaging method for localizing and characterizing thoracic endometrial lesions [121]. Lung nodules represent the least common manifestation of thoracic endometriosis, occurring in only about 6% of cases [119]. In thoracic endometriosis, a chest CT obtained during menstruation may show parenchymal ground-glass opacities, with or without nodules, which, when considered alongside a characteristic history and symptoms, can support the diagnosis of pulmonary endometriosis. Comparing scans taken during and outside the menstrual period can help identify pulmonary hemorrhage and catamenial changes, while a post-menstrual CT can better define any nodular lesion and confirm resolution of the hemorrhage [120,121,122].

Management of thoracic endometriosis primarily aims to suppress ovarian steroid hormone production, with gonadotropin-releasing hormone (GnRH) analogs as first-line therapy. A well-documented example by Aboujaoude et al. describes a 34-year-old woman with catamenial hemoptysis and prior pelvic endometriosis, in whom pulmonary endometriosis was diagnosed based on symptom timing and CT findings. She was treated exclusively with the gonadotropin-releasing hormone agonist triptorelin pamoate for six months, achieving complete radiologic resolution and remaining symptom-free for nine months, demonstrating that selected cases may be successfully managed with medical therapy alone [120]. Alternatives include oral contraceptives, progestins, danazol, aromatase inhibitors, and GnRH antagonists, with no proven difference in efficacy. Discontinuation of hormonal suppression is associated with a high recurrence risk. Surgical management is reserved for refractory or recurrent disease and often requires a multidisciplinary approach combining video-assisted thoracoscopic surgery (VATS) for thoracic lesions and laparoscopy for concomitant pelvic disease. Postoperative hormonal suppression is recommended to minimize recurrence [115].

## 17. Parasitic and Other Uncommon Infectious Causes of Hemoptysis

### 17.1. Leech Infestation

Live foreign body or parasite, especially in the oro- or nasopharynx, is generally a rare entity but a common emergency presentation in some parts of the world, particularly where access to safe water is limited. Leeches are hermaphroditic parasites that vary in shape and size, ranging from elongated and cylindrical to broad or ovoid, and can reach up to 45 cm in length. They possess muscular suckers at both their anterior and posterior ends. Their presence in body cavities is known as internal hirudiniasis, which is associated with drinking from or swimming in contaminated spring waters. Leech infestation is more common among individuals of lower socioeconomic status. A leech is a rare foreign body that adheres to the mucosa and can be located anywhere in the upper respiratory tract. Depending on its location, patients may experience various symptoms such as epistaxis, nasal obstruction, a sensation of a moving foreign body, hemoptysis, dyspnea, voice changes, and a feeling of airway compromise. Due to anesthetic agents found in leech saliva, some patients may not perceive the leech as a foreign body or may not even feel it while it is attached to the mucosa. Based on its site of attachment, leeches may cause problems such as continuous bleeding, hematemesis, nosebleeds, hemoptysis, and an irritable cough. If the parasite reaches the larynx or trachea, it may cause life-threatening complications such as stridor or even fatal airway obstruction [123,124,125].

Management after diagnosis can be achieved in several ways. Due to the leech’s soft and slippery body, removal with forceps may be challenging and can sometimes result in partial transection of the parasite. In some cases, heated instruments have been used for extraction, although hypertonic saline or local anesthetic solutions are more commonly applied to cause paralysis and facilitate detachment. Once the parasite is completely removed, symptoms usually resolve promptly [123,126].

### 17.2. Paragonimiasis (Lung Fluke Infection)

Paragonimiasis is a parasitic infection caused by *Paragonimus* species, also known as lung flukes. It is primarily encountered in endemic regions and is typically associated with the ingestion of raw or inadequately cooked freshwater crustaceans. Following ingestion, the larvae are released in the gastrointestinal tract and subsequently migrate through the peritoneal cavity and diaphragm to reach the lungs [127,128].

Although infection can be asymptomatic, there are reported cases of early pulmonary paragonimiasis presented as chest pain, cough and dyspnea. Chronic infection occurs when mature lung flukes inhabit the lung and it is characterized by recurrent hemoptysis. Laboratory evaluation may reveal eosinophilia, while imaging can detect migratory pulmonary infiltrates. Radiographic findings include ring-shaped opacities with adjacent linear streaks as a result of burrowing, pleural thickening and mass lesions. These findings are often mistaken for tuberculosis, particularly in endemic regions. Lung lesions are located peripherally and are more common in mid-lung and lower lung zones, although Yadav et al. reported a case with upper-lobe involvement [127,129].

Diagnosis is most often confirmed by identifying *Paragonimus* eggs in sputum or bronchoalveolar lavage specimens. In some cases, surgical intervention is required, as illustrated by Boé et al., who reported a patient in whom transbronchial biopsy was not conclusive and revealed non-necrotizing granulomas within the bronchial wall and moderate chronic inflammation with abundant eosinophils. The patient subsequently underwent segmentectomy, and histopathological examination demonstrated necrotizing granulomatous inflammation containing parasitic ova consistent with *Paragonimus* species. Once diagnosed, patients are usually treated with praziquantel, which typically results in complete resolution of symptoms [127,130].

### 17.3. Pulmonary Hydatid Cyst

Echinococcosis is a parasitic infection caused by the larval stage of cestodes of the genus *Echinococcus*. After ingestion, the eggs hatch in the small intestine, releasing embryos that invade the mucosa and hematogenously spread to organs where the cyst develops. The liver is affected in over 65% of cases, followed by the lungs in about 25%. In most cases, lesions are solitary, but multiple cysts or multiple organ involvement have also been reported. Due to their slow growth, cysts often remain asymptomatic for years and may present in adulthood, even if acquired in childhood [131,132].

Pulmonary hydatidosis with cysts larger than 5 cm may cause bronchial compression. Complications of this condition include cyst rupture, secondary infection, suppuration, and pneumothorax. Rupture of the cyst can present with symptoms such as chest pain, cough, fever, salty taste in the mouth, blood coughing. Hemoptysis is a common manifestation of rupturing cyst, and in some cases, such as the one Tekinbas et al. [133] presented, it can be massive and life-threatening. Another life-threatening complication of rupturing hydatid cyst is anaphylaxis accompanied by urticaria and wheezing due to hypersensitivity to the cyst fluid [133,134,135].

Chest radiography is usually the initial imaging method and typically reveals sharply defined, round-to-oval homogeneous opacities of variable size and number. Computed tomography of the chest is a superior modality, demonstrating cysts with smooth walls and predominantly homogeneous internal contents of water or near-water density [131,134].

Radiologic signs of ruptured hydatid cyst include several characteristic appearances, such as the double-arch (Cumbo), iceberg, rising sun, serpent, and whirl signs. When air enters the cyst, the endocyst may collapse, producing an air–fluid level. Floating membranes on the fluid surface create the classic “water lily” (or Camelot) sign. In cases where all parasitic contents are evacuated, only the host-derived pericyst remains, presenting as the “empty cyst” sign [136].

Surgery remains the gold standard treatment for pulmonary hydatid cysts of any size, although some patients may be managed pharmacologically with oral benzimidazoles such as mebendazole or albendazole, but in such cases long-term follow-up is required [131].

## 18. Conclusions

Despite significant progress in diagnostic procedures, a certain proportion of hemoptysis cases still remain idiopathic, with no identifiable cause [2]. The responsibility lies with clinicians to persist in searching for an etiology, to remain open to rare and unexpected entities, and to continuously inform and educate themselves in order to provide the best possible care for patients and to ensure prompt action in potentially life-threatening situations. The examples reviewed in this article illustrate how diverse the potential causes and therapeutic approaches can be, reminding us that hemoptysis is not a uniform clinical problem but rather a multifaceted syndrome requiring careful and individualized evaluation.

## Figures and Tables

**Table 1:** Rare causes of hemoptysis discussed in this review.

Cause/Entity	Main Category	Typical Clinical Context
Systemic arterial vascularization of the lung	Congenital vascular	Hemoptysis, dyspnea, pulmonary hypertension
Pulmonary artery hypoplasia/agenesis	Congenital vascular	Recurrent infections, hemoptysis
Pulmonary arteriovenous malformations (PAVMs)	Vascular malformation	Hemoptysis, shunt symptoms
Systemic–pulmonary arterial fistulas	Vascular anomaly	Recurrent/massive hemoptysis
Aortobronchial fistula	Vascular	Massive hemoptysis
Rasmussen aneurysm	TB-related vascular	Massive hemoptysis
Hughes–Stovin syndrome	Systemic vasculitis	Hemoptysis with thrombosis
Diffuse alveolar hemorrhage (DAH)	Microvascular	Hemoptysis, anemia, infiltrates
Idiopathic pulmonary hemosiderosis	DAH subtype	Recurrent hemoptysis
Targeted/immunotherapy-related DAH	Treatment-related	Hemoptysis or respiratory failure
Drug-induced lung injury	Toxic	Hemoptysis after inhalation exposure
Burn/inhalation injury	Toxic	Airway injury, hemoptysis
Bronchial Dieulafoy’s disease	Vascular anomaly	Massive hemoptysis
Tracheo-innominate fistula	Iatrogenic	Massive bleeding
Carotid–tracheal fistula	Iatrogenic	Massive hemoptysis
Tracheal granulation tissue	Iatrogenic	Delayed hemoptysis
Pulmonary hamartoma	Benign tumor	Hemoptysis (rare)
Foreign body aspiration	Mechanical	Cough, hemoptysis
Broncholithiasis	Structural	Chronic cough, hemoptysis
Lipoid pneumonia	Inflammatory	Chronic cough, rare hemoptysis
Pulmonary endometriosis	Hormonal	Catamenial hemoptysis
Leech infestation	Parasitic	Bleeding, airway symptoms
Paragonimiasis	Parasitic	Chronic hemoptysis
Hydatid cyst	Parasitic	Hemoptysis after rupture

**Table 2:** Suggested diagnostic approach to rare causes of hemoptysis.

Step	Clinical Scenario	Suggested Evaluation
1	Initial presentation of hemoptysis	Clinical assessment, laboratory tests, chest X-ray
2	Suspicion of common causes	Chest CT ± bronchoscopy (to further evaluate common causes)
3	No clear etiology after initial work-up	Contrast-enhanced CT/CT angiography
4	Recurrent or unexplained hemoptysis	Bronchoscopy with BAL
5	Identified or suspected vascular source	Angiography (diagnostic and therapeutic)
6	Systemic symptoms present	Immunological testing (vasculitis, DAH)
7	Relevant exposure history	Targeted investigations (drugs, parasites, aspiration)
8	Specific clinical contexts	Tailored evaluation (e.g., catamenial hemoptysis, tracheostomy complications, therapy-related toxicity)

Abbreviations: CT, computed tomography; BAL, bronchoalveolar lavage; DAH, diffuse alveolar hemorrhage.

## Data Availability

Data is contained within the article.

## Abbreviations

BAL	bronchoalveolar lavage;
CT	computed tomography;
CTA	computed tomography angiography;
DAH	diffuse alveolar hemorrhage;
HHT	hereditary hemorrhagic telangiectasia;
IPH	idiopathic pulmonary hemosiderosis;
TES	thoracic endometriosis syndrome.
